# A Multi-Label Learning Based Kernel Automatic Recommendation Method for Support Vector Machine

**DOI:** 10.1371/journal.pone.0120455

**Published:** 2015-04-20

**Authors:** Xueying Zhang, Qinbao Song

**Affiliations:** Department of Computer Science & Technology, Xi'an Jiaotong University, 28 Xian-Ning West Road, Xi'an, Shaanxi 710049, P. R. China; New Jersey Institute of Technology, UNITED STATES

## Abstract

Choosing an appropriate kernel is very important and critical when classifying a new problem with Support Vector Machine. So far, more attention has been paid on constructing new kernels and choosing suitable parameter values for a specific kernel function, but less on kernel selection. Furthermore, most of current kernel selection methods focus on seeking a best kernel with the highest classification accuracy via cross-validation, they are time consuming and ignore the differences among the number of support vectors and the CPU time of SVM with different kernels. Considering the tradeoff between classification success ratio and CPU time, there may be multiple kernel functions performing equally well on the same classification problem. Aiming to automatically select those appropriate kernel functions for a given data set, we propose a multi-label learning based kernel recommendation method built on the data characteristics. For each data set, the meta-knowledge data base is first created by extracting the feature vector of data characteristics and identifying the corresponding applicable kernel set. Then the kernel recommendation model is constructed on the generated meta-knowledge data base with the multi-label classification method. Finally, the appropriate kernel functions are recommended to a new data set by the recommendation model according to the characteristics of the new data set. Extensive experiments over 132 UCI benchmark data sets, with five different types of data set characteristics, eleven typical kernels (Linear, Polynomial, Radial Basis Function, Sigmoidal function, Laplace, Multiquadric, Rational Quadratic, Spherical, Spline, Wave and Circular), and five multi-label classification methods demonstrate that, compared with the existing kernel selection methods and the most widely used RBF kernel function, SVM with the kernel function recommended by our proposed method achieved the highest classification performance.

## Introduction

The Support Vector Machine (SVM) was originally proposed for solving binary classification problems by Cortes and Vapnik [1, 2], and then extended by Hsu and Lin [3, 4] for dealing with multi-class classification problems via constructing one binary classifier for each pair of distinct classes. Based on the Vapnik-Chervonenkis (VC) dimension theory [5] and structural risk minimization (SRM) [6], SVM has been successfully applied to address small sample, nonlinear and high dimensional learning problems such as text categorization [7–9], pattern recognition [10–12], time-series prediction [13, 14], gene expression profile analysis [15–17], and protein analysis [4, 18].

SVM classifies the data objects via identifying the optimal separating hyperplanes among classes. Determining a class boundary in the form of a separating hyperplane is adequate for simpler cases where the classes are nearly or completely linearly separable. However, in practice, classes are usually complexity, high dimensionality or not linearly separable, so a kernel function is employed to project original data into a higher dimensional space at first, and then a linear separating hyperplane with the maximal margin between two classes are constructed [1].

According to the Reproducing Kernel Hilbert Spaces (RKHS) [19, 20], the kernel function, which is represented as a legitimate inner product *K*(*u*, *v*) = (*ϕ*(*u*) ⋅ *ϕ*(*v*)), can be any positive definite function that satisfies the Mercer conditions [2]. While most commonly used kernel functions are linear, polynomial, radial basis function and sigmoid function, there are many other complicated kernel functions derived by aggregating multiple base kernel functions. In essence, the generalization capacity of SVM depends on the choice of kernel function and the setting of misclassification tolerance parameter *C*, in which the *C* is directly related to certain kernel [21, 22]. Hence, a careful choice of the kernel function is primary for SVM in order to produce an appropriate classification boundary.

For the purpose of determining a optimal kernel function, most researchers devote to tweaking the associative parameters for a specified kernel function via trial-and-error, less on selecting an appropriate kernel function. Generally, the existing methods can be divided into four categories (1) cross-validation [23–25] is most commonly used to find an optimal kernel for a new data; (2) multiple kernel learning (MKL) [26, 27] attempts to construct a generalized kernel function so as to solve all classification problems through combing different types of standard kernel functions; (3) genetic programming [28, 29] uses Gene Expression Programming algorithms to evolve the kernel function of SVM; and (4) automatic kernel selection method with C5.0 [30, 31] aims to recommend a special kernel function for different classification problems based on the statistical data characteristics and distribution information.

Apart from cross-validation, MKL and genetic programming methods require numerous iterations for converging towards a reasonable solution [32], all existing methods are trying to seek a single optimal kernel function in terms of classification accuracy. However, for some kernels, although the difference of classification accuracy is minor, the differences of the corresponding number of stored support vectors or the time complexity [33] can be significant. This means the selected kernel may not be the best one. For instance, suppose the classification accuracy of SVM with kernel function A is slightly higher than that with kernel function B, and the former costs much more time than the latter. Usually, kernel function A is selected as the best one. However, this is not the best option and kernel function B should be more appropriate for practical applications. Therefore, it is fallacious to evaluate the performance of SVM with one kernel function just in terms of classification accuracy, and further selection should be proceeded to pick out those applicable kernels in the light of overall performance. Noting that, due to the balance between classification accuracy and CPU time, SVM might perform equally well with different kernels on a same classification problem. In this case, kernel selection can be viewed as a multi-label learning problem so that an applicable kernel set for a new problem with satisfied classification performance can be recommended.

In the multi-label learning, multi-label classification (MLC) [34–36] has been widely applied in semantic annotation [37, 38], tag recommendation [39], rule mining [40], and information retrieval [41, 42]. Recently, multi-label classification has been studied and adopted for recommending an applicable set of classification algorithms by Wang, et.al [43]. Inspired by their research work, we proposed a new multi-label meta-learning based kernel recommendation method this paper presents, in which data sets are described by the corresponding characteristics and their corresponding applicable kernel sets are identified in terms of the adjusted ratio of ratios (*ARR*) [44] via cross-validation and the relationship between them is discovered by multi-label classification algorithms and further used to recommend applicable kernels for new problems. Extensive experiments over 132 UCI benchmark data sets, with five types of data set characteristics, eleven commonly used kernels (Linear, Polynomial, Radial Basis Function, Sigmoidal function, Laplace, Multiquadric, Rational Quadratic, Spherical, Spline, Wave and Circular), and five multi-label classification methods demonstrate that, compared with the existing kernel selection methods and the most widely used RBF kernel function, SVM with the kernel function recommended by our proposed method achieved a higher classification performance.

The remainder of the paper is set up as follows. The related work is briefly reviewed in Section Previous Work. The proposed method is concretely introduced in Section Multi-label Learning Based Kernel Recommendation Method. The experimental process and the result analysis are provided in Section Experimental Study. Finally, the conclusion of our work is drawn in Section Conclusion.

## Previous Work

In the past decades, the issue of kernel selection for SVM has attracted much attention and lots of methods have been proposed. Most research work concentrated on the parameter optimization for a pre-specified kernel function [45–48] via cross-validation, exhaustive grid search or evolutionary algorithms, etc, whereas less on kernel selection. Generally, the state-of-the-art kernel selection methods can be categorized into four classes: cross-validation, multiple kernel learning, evolutionary methods, and meta-learning based methods.

Cross-validation is the most frequently used method for model selection, the problem is that the computational cost is too much to be used in practice since the learning problem must be iterated *n* times. For SVM, the optimal kernel is usually achieved after minimizing the *n*-fold cross-validation error (i.e. the leave-one-out classification error) [23–25].

Multiple kernel learning methods (MKL) [32, 33, 49] are the linear or nonlinear combination of different kernels instead of a single kernel function. Since different kernels correspond to different notions of similarity and they may be using inputs of different representations possibly from different sources or modalities, combining kernels is one possible way to combine multiple information sources. This type of methods aim to yield a general kernel function for solving any problem. However, in fact, it is difficult to determine which kernels should be combined and it converges very slowly for a big data set.

Evolutionary kernel selection methods use the *n*-fold cross-validation accuracy as the fitness criterion. Howley et al. [50] and Sullivan et al. [29] attempted to find the near-optimal kernels for SVM using genetic programming system, in which the kernel functions are represented as trees, input variables or numerical constants are represented as the leaves and their values are passed to nodes. It performed some numerical or program operations before passing on the result further towards the root of the tree, where the classification error and “tiebreaker” are taken as the fitness function. Kanchan et al. [28] employed the Gene Expression Programming to train a SVM with the most suitable kernel function, where the cross-validation accuracy is calculated for measuring the fitness of a kernel. These methods show wide applicability, but the combined computational overhead of genetic programming and SVM remains a major unresolved issue.

Meta-learning based kernel selection method [30] applied decision tree to generate the associate rules between the most appropriate kernel and data set characteristics for support vector machine. In this approach, three types of measures (classical, distance and distribution-based statistical information) are collected for characterizing each data set, and the classification accuracy is used to evaluate the performance of SVM with the selected kernel function. Similarly, Wang et al. [31] proposed to assign a suitable kernel function for a given data set after discerning its approximate distribution with PCA [51].

The first three types of methods would result in a large number of evaluations and unacceptable CPU runtime, it is unpractical for solving the real classification tasks. In addition, the existing automatic kernel selection methods pay more attention on how to create or optimize a promising kernel for a given classification problem, less on kernel selection from amounts of available kernel functions. For dealing with issue that multiple kernels might perform equally well on a given data set, contrast to the multiple kernel learning methods, we view kernel selection as a multi-label classification problem and propose a multi-label learning based kernel recommendation method to identify all the applicable kernels for different classification problems.

## Multi-label Learning Based Kernel Recommendation Method

In this section, we concretely introduce the fundamental of our multi-label learning based kernel recommendation method. We first give an overview of the proposed method in subsection General View of the Method, and then describe each component of our recommendation method in subsection Meta-knowledge Database Generation and subsection Model Construction and Recommendation, respectively.

### General View of the Method

**Fig 1:**
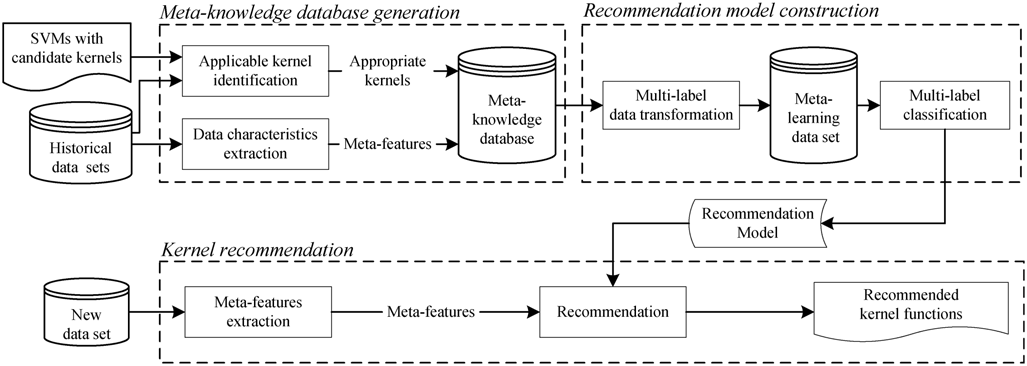
Framework of the kernel recommendation method.

As we know, for different data sets, the performance of SVM with a specific kernel function can be different. This means that, just as there is a dual relation between data set characteristics and the performance of a classification algorithm [52, 53], there also exists a relationship between data set characteristics and the performance of a kernel function when SVM is used as a classification algorithm. Thus, before recommending a suitable kernel function for a classification problem, this relationship must be modeled. Furthermore, in order to build this relationship, the characteristics of a data set and the corresponding performance of the appropriate kernel function(s) should be obtained. Therefore, our proposed method consists of three parts: meta-knowledge database generation, recommendation model construction, and kernel recommendation. Fig 1 shows the details.

1. *Meta-knowledge database generation*. This preparation stage creates a meta-knowledge database based on the historical data sets and all the possible kernel functions. Specifically, for each historical data set, the characteristics measures are extracted as the meta-features, while the corresponding applicable kernel set are identified as the meta-targets through constructing and evaluating SVM with each candidate kernel. After that, the meta-knowledge database is created by merging the meta-features and the applicable kernel set for each of the historical data sets.
2. *Recommendation model construction*. At this stage, based on the meta-knowledge database, multi-label classification algorithms are applied to the meta-knowledge data consisting of meta-features and meta-targets, and the recommendation model is built.
3. *Kernel recommendation for the new data set*. When recommending kernels for a new problem, its characteristics are extracted and passed to the recommendation model, the output of the model is the suitable kernel functions for the new problem.

### Meta-knowledge Database Generation

Meta-knowledge database captures the knowledge about which kernels perform well on what data sets when SVM is used as the classification algorithm. In this section, we first introduce all the measures for characterizing a data set, and then we explain how to determine the applicable kernel set for each data set.

#### Meta-features

The meta-features consist of measures extracted from data sets for uniformly depicting data set characteristics. Pavel et al. [54] first proposed to generate a set of rules for characterizing the applicability of classification algorithms using meta-level learning (ML). Afterwards, Shawkat and Kate [53] presented a rule-based classification selection method, which built on the data characteristics, to induce which types of algorithms are appropriate for solving which types of classification problems, and then they explored a meta-learning approach to automatic kernel selection for support vector machines [30]. Overall, the above mentioned work characterized data sets by the simple, statistical and information theory based measures. These measures are not only conveniently and efficiently calculated, but also related to the performance of machine learning algorithms [55, 56].

Up to now, data measures are not limited to statistical or information theoretic descriptions. Recently, some other well-established measures have been developed in different perspectives as well, such as problem complexity measures, Landmarking measures, model-based measures and structural measures. Table 1 describes each type of data characteristic measures in detail.

- Problem complexity measures
  Ho and Basu [57] explored a number of measures to characterize the difficulty of a classification problem, focusing on the geometrical complexity of the class boundary. These problem complexity measures can highlight the manner in which classes are separated or interleaved, a factor that is most critical for classification accuracy.
- Landmarking measures
  Bensusan et al. [58–60] introduced the meta-learning by landmarking various learning algorithms. The main idea of landmarking is using some simple and efficient learning algorithm themselves to determine the location of a specific learning problem, which is represented by the disagreement pattern between a set of standard classifiers. The disagreement patterns not only point towards different types of classification problems, but also indicate the novelty and the usefulness of a classifier with respect to a set of classification problems and classifiers. In our study, we build a small set of standard classification algorithms (Naive Bayes, IB1 and C4.5) on each data set and then record their classification performance as the Landmarking measures.
- Model based measures
  Peng et al. [61] presented new measures to capture the characteristics from the structural shape and size of the decision tree induced from a given data set. We employed C5.0 tree algorithm to construct the standard decision tree on each data set, and then obtained a total of 15 measures for describing the properties of each decision tree.
- Structural and statistical information based measures
  Song et al. [62] presented the structural and statistical information based data set characteristics, and used for constructing a recommendation method for classification algorithms. Firstly, the given ordinary data set is transformed into the corresponding binary data set; then a one-item set *V*_*I*_ and a two-item set *V*_*II*_ are extracted, where *V*_*I*_ captures the distribution of the values of a given attribute while *V*_*II*_ reflects the correlation between two features; finally, to achieve unified representation and comparison of different data sets, both *V*_*I*_ and *V*_*II*_ are sorted in ascending order and then a unified features set is generated by computing the statistical summary of both items, including their minimum, seven octiles and the maximum. Compared with the traditional data set characteristic measures, this type of measures is confirmed to be superior to the others.

**Table 1:** Data characteristic measures.

Simple, statistical and information theoretical measures		Problem complexity measures	
Notation	Measures	Notation	Measures
F	Number of features	BL	Length of class boundary
OL	Number of outliers	1NN	Nonlinearity of 1NN classifier
I	Number of instances	Fisher	Maximum Fisher’s discriminant ratio
C	Number of class labels	NLP	Nonlinearity of linear classifier by LP
MV	Number of missing values	AP	Average number of points per dimension
*F*_*bin*_	Number of binary features	RS	Percentage of retained adherence subsets
MI	Number of missing instances	RNN	Ratio of average intra/inter class nearest neighbor distances
*F*_*nom*_, *F*_*num*_	Number of nominal and numeric features		
*C*_*maj*_, *C*_*min*_	Percentage of majority and minority class		
Prc	Prctile		
*V*	Variance	Model-based measures	
*k*	Kurtosis		
*s*	Skewness	Notation	Measures
TM	Trim mean	TW	width of tree
HM	Harmonic mean	TH	height of tree
GM	Geometric mean	Nodes	number of nodes
CG	Center of gravity	Leaves	number of leaves
IQR	Interquartile range	longBranch	length of longest branches
*std*	Standard deviation	shortBranch	length of shortest branches
CC	Canonical correlation	minAttr	minimum occurrence of attributes
*r*	Correlation coefficient	meanBranch	mean of the length of each branch
*Mad*	Mean absolute deviation	maxAttr	maximum occurrence of attributes
NSR	Noise-signal ratio	maxLevel	maximum number of nodes at one level
*H*(*C*)	Entropy of classes	meanLevel	mean of the number of nodes at one level
*H*(*X*)	Mean entropy of variables	devLevel	standard deviation of the number of nodes
ENV	Equivalent number of variables	devBranch	standard deviation of the length of each branch
*E*_*max*_, *E*_*min*_	Maximum and minimum eigenvalue	meanAttr	mean of the number of occurrences of attributes
$\bar{H}(C,X)$	Mean mutual entropy of class and variables	devAttr	standard deviation of the number of occurrences of attributes

#### Applicable Kernels Identification

Applicable kernels are kernels among which there is no significant differences in classification performance of SVM. For the objective of modeling the relationship between data set characteristics and applicable kernel functions, it is essential to identify an applicable kernel set for each historical data set as the target concept.

The identification of applicable kernels can be briefly described below: for each historical data set, SVM with each candidate kernel is adopted to classify each historical data set, and then the applicable kernel set of each data set is identified via evaluating and comparing the classification performances of SVM with all candidate kernels.

When evaluating the performance of SVM with two different kernel functions on a given data set, there may not be large difference in terms of classification accuracy, but there may be significant difference between two different kernels in the number of stored support vectors or training time complexity [33]. For example, if SVM with kernel A is slightly better than that with kernel B in success ratio but SVM with kernel A takes much more CPU time than that with B, then it is more possible that kernel B is chosen as the kernel function. Thus, taking the success ratio of classification and CPU time into consideration when evaluating the performance of SVM with one special kernel function is rational. Moreover, the issue of class imbalance distribution often exists in real-world applications, in this case, classification accuracy is not a powerful metric used for reflecting the performance of SVM with different kernel functions. Alternatively, the area under the curve (AUC) [63] is theoretically and empirically validated to be more suitable for evaluating the performance of a classification algorithm under this situation. Thus, the adjusted ratio of ratios (*ARR*) [44, 64] is modified and adopted as multi-criteria measure for evaluating the classification performance of SVM with different kernels, which aggregates information concerning the success ratio AUC and CPU time of SVM with each candidate kernel and even realizes the compromise of both.

The *ARR* of SVM with the kernel function *k*_*p*_ on the data set *d*, $ARR_{k_{p}}^{d}$ is defined as:

$$\begin{array}{ccc} ARR_{k_{p},k_{q}}^{d} & = & \frac{\frac{AUC_{k_{p}}^{d}}{AUC_{k_{q}}^{d}}}{1+\beta \times log\left(\frac{T_{k_{p}}^{d}}{T_{k_{q}}^{d}}\right)},\\ ARR_{k_{p}}^{d} & = & \frac{\sum_{q=1}^{m}ARR_{k_{p},k_{q}}^{d}}{m}. \end{array}$$ (1)

Where $AUC_{k_{p}}^{d}$ and $T_{k_{p}}^{d}$ represent AUC and CPU time of kernel *k*_*p*_ on data set *d*, respectively, *k*_*q*_ denotes each of other kernels, and *β* represents the relative importance of AUC and CPU time, it is in the range of [0, 1] and often defined by users.

For the purpose of obtaining a stable classification performance of SVM with different kernel functions and making full use of the historical data set, a 10×10-fold cross-validation is employed. This means the 10-fold cross-validation is repeated 10 times for the SVM with a given kernel function on each data set with different random seeds, and the effects from the order of inputs are reduced. The detailed procedure for evaluating the performance of kernels is shown in Table 2.

**Table 2:** Algorithm 1. PerformanceEvaluation().

	**input**: *data*—a given historical data set;
	SVM—the default classifier;
	*allKernels*—a set of candidate kernels;
	**output**: *ARR*—the classification performance of SVM with a specific kernel;
1	*TIMES* = 10; *FOLDS* = 10; *M* = sizeof(*allKernels*);
2	**for** *t* = 1 to *TIMES* **do**
3	randomize the order of *data*;
4	generate *FOLDS* bins from *data*;
5	**for** *i* = 1 to *FOLDS* **do**
6	TestData = bins[*i*];
7	TrainingData = *data*-TestData;
8	**for** *m* = 1 to *M* **do**
9	(*predictor*[*m*], Time[*i*][*m*]_*training*_) = SVM(TrainingData, *m*);
10	(AUC[*i*][*m*], Time[*i*][*m*]_*test*_) = apply *predictor*[*m*] to TestData;
11	Time[*i*][*m*] = Time[*i*][*m*]_*training*_ + Time[*i*][*m*]_*test*_;
12	**for** *m* = 1 to *M* **do**
13	ARR[*i*][*m*] = $getARR_{m}^{d}$(AUC[*i*], Time[*i*], *m*);
14	return *ARR*;

Once the performance array, in which each column represents the *ARR* performance of SVM with one special kernel function and each row denotes each fold cross-validation with *M* kernel functions, of SVM with all candidate kernel functions is obtained, for a given data set, a multiple comparison procedure (MCP) [65] is used to identify the applicable kernel set that consists of the top-scoring candidate kernels without significant performance differences among them. Since the value distribution of performance is unknown, the simple, yet safe and robust non-parametric tests are used to statistically comparing classifiers. Specially, the Friedman test [66, 67] with Holm’s procedure [68] is employed to compare multiple kernel functions on each given data set. The Friedman test is first employed to check whether there is significant difference between all candidate kernels at the significant level *α* = 0.05. The null hypothesis is *H*_0_ : *k*_1_ = *k*_2_ = … = *k*_*m*_, which states that all kernels are equivalent, if the test result *p* < 0.05, then the null-hypothesis is rejected and the post-hoc test Holm-Procedure is proceeded to find out those kernels outperforming others, or there is no significant difference among all kernels. Table 3 provides the details of the applicable kernels identification for a given data set.

**Table 3:** Algorithm 2. ApplicableKernelIdentification().

	**input**: *data*—a given historical data set;
	*allKernels* = *k*_1_, *k*_2_, …, *k*_*m*_—the set of all candidate kernels;
	**output**: *appKernels*—a set of applicable kernels;
1	ARR = *PerformanceEvaluation*(*data*, *allKernels*, SVM);
2	*H*_0_ : *k*_1_ = *k*_2_ = … = *k*_*m*_;
3	*p* = Friedman(ARR, *m*, 0.05);
4	**if** *p* < 0.05 **then**
5	// *H*_0_ is rejected;
6	*appKernels* = HolmProcedure(ARR, *m*, 0.05);
7	**else**
8	// *H*_0_ is accepted;
9	*appKernels* = allKernels;
10	**return** *appKernels*;

### Model Construction and Recommendation

In this section, we elaborate the process of recommendation model construction. Firstly, we introduce the state-of-the-art multi-label classification methods used for modeling the relationship between data set characteristics and the performance of different kernels in subsection Multi-label Classification. Secondly, we present the multi-label feature selection methods that used to exclude useless features affecting the construction of recommendation model in subsection Multi-label Feature Selection. Finally, we provide the construction method of the multi-label learning based kernel recommendation model in subsection Multi-label Kernel Recommendation Model Construction, and give the measures used to evaluate the performance of our recommendation method in subsection Multi-label Evaluation Metrics.

#### Multi-label Classification

Traditional single-label classification is concerned with learning from a set of examples associated with a single class label from a set of disjoint labels, and the applicable kernels for a classification problem usually are not only one. Moreover, multiple-label classifications [34, 36, 69] learn the problems where each example is associated with more than one class labels. Therefore, the kernel recommendation is a multi-label classification problem. Two main categories of multi-label classification methods can be used to build kernel recommendation model, they are problem transformation and algorithm adaption.

- Problem transformation
  The purpose of problem transformation is to convert a multi-label learning problem into a traditional single-label classification problem by the methods listed below: (1) subjectively or randomly select one of the multiple labels for each multi-label instance and discard the rest; (2) simply discard every multi-label instance from the multi-label data set and only retain those instances with single label; (3) Label powerset (LP) considers each different set of labels in the multi-label data set as a single label classification task; (4) Include labels classifier (ILC) decomposes each example (*x*, Y) into ∣*L*∣ examples (*x*, *l*, *Y*[*l*]), for all *l* ∈ *L*, where *Y*[*l*] = 1 if *l* ∈ *Y*, and *Y*[*l*] = −1 otherwise; (5) Binary relevance (BR) learns *L* binary classifiers, one for each different label in *L*. It transforms the original data set into ∣*L*∣ data sets *D*_*l*_ that contain all examples of the original data set, labeled positively if the label set of the original example contained *l* and negatively otherwise. For the classification of a new instance, it outputs the union of the labels *l* that are positively predicted by the ∣*L*∣ classifiers; and (6) Calibrated label ranking (CLR) introduces a calibration label representing the boundary between relevant and irrelevant labels, and effectively produces an ensemble combining the models learned by the conventional binary relevance ranking approach and the pairwise comparison approach.
- Algorithm adaptation
  Unlike the problem transformation, the objective of algorithm adaptation is to modify the existing single-label classification algorithms, and then adapt them to solve the multi-label classification problems. The prevalent algorithms contain C5.0 [70], BoosTexter [71], the multi-label *k*-nearest neighbor ML-KNN [72], the multi-label kernel method RANK-SVM [73], the multi-class multi-label neural networks BP-MLL [74], and MMP [75]. The first three methods are the most commonly used in multi-label learning.
  Clare and King [70] adopted C4.5 to handle the multi-label biological problems through modifying the definition of entropy, its output is a decision tree or equivalently a set of symbolic rules allowing to be interpreted and compared with existing biological knowledge. However, it just learns the rules for biological interest rather than predict all examples.
  Schapire and Singer [71] proposed a Boosting-based system for text categorization (BoosTexter) on the basis of the popular ensemble learning method ADABOOST [76]. In the multi-label training phase, BoosTexter maintains a set of weights over training examples and labels. As boosting progresses, training examples and their corresponding labels that are hard to predict correctly get incrementally higher weights, while examples and labels that are easy to classify get lower weights.
  ML-KNN [72] is a multi-label lazy learning approach, which is derived from the traditional *k*-nearest neighbor (*k*NN) algorithm. Concretely, for each unseen instance, its *k* nearest neighbors are first identified in the training set. After that, based on the number of neighboring instances belonging to each possible class, the label set for the unseen instance is determined by maximum a posteriori (MAP) principle. Experimental results confirmed that ML-KNN slightly outperforms BoosTexter, and is far superior to ADABOOST.MH and RANK-SVM. Thus, ML-KNN has been applied to solve the real-world multi-label learning problems.

Aiming to construct our multi-label learning based kernel recommendation method as well as possible, we select several representative and effective multi-label classification methods in the level of problem transformation and algorithm adaption to build the recommendation model, respectively.

#### Multi-label Feature Selection

Feature selection can provide more suitable features for building classification models. Considering single-label feature selection methods dedicate to filter out useless and redundancy features for single-label based learning while our kernel recommendation is a multi-label learning based method, before constructing the recommendation model, multi-label feature-selection techniques are applied to select those critical meta-features for model construction. The multi-label feature-selection techniques can be classified into external and internal strategies.

Internal strategy [77] aims to utilize the multi-label statistical relationship such as document-label information and label-label relationships within the design of feature selection algorithms. On the contrary, external strategy [78] transforms multi-label training data into single-label data before feature selection, so the traditional single-label feature selection algorithms can be applied. In this section, we mainly introduce the external strategy since some previous single-label feature selection methods are applicable for multi-label problems with high efficiency and effectiveness.

Yang and Pedersen [79] made a comparative study on five popular feature selection methods and confirmed that Information Gain (*IG*) and CHI-SQUARE (*CHI*) are the most effective and comparable. Here, besides *IG* and *CHI*, we also apply Relief [80] to provide suitable features for building kernel recommendation model. Relief is a practical feature selection method that evaluates the worth of a feature by repeatedly sampling an instance and considering the values of the given feature for the nearest instance belong to the same and the different classes. *IG* [79] evaluates the worth of a feature by computing the information gain related to the class. If the information gain of a feature is less than the predetermined threshold, then the feature will be removed from the feature space. *CHI* [79] evaluates the worth of a feature by computing the value of the chi-squared statistic with respect to the class. The chi-squared statistic is a normalized value and it is comparable across features of the same class.

#### Multi-label Kernel Recommendation Model Construction

In order to thoroughly explore our proposed kernel recommendation method and adequately make use of the available data, five multi-label classification algorithms and three multi-label feature selection methods are employed to build kernel recommendation model via the jackknife cross-validation technique. The detail is shown in Table 4.

**Table 4:** Algorithm 3. ModelConstruction().

	**input**: *metaDB*—the meta-knowledge data base;
	*FS* = {*Relief*, *IG*, *CHI*}—the set of multi-label feature selection methods;
	*MLC* = {*BR*, *LP*, *CLR*, *ILC*, *ML-KNN* }—the set of multi-label classification methods
	**output**: recPerformance—the performance of the multi-label based kernel recommendation model;
1	*N* = *sizeof*(*metaDB*);
2	**for** *ml* ∈ *MLC* **do**
3	**for** *fs* ∈ *FS* **do**;
4	**for** *i* = 1 ∈ *N* **do**;
5	//for the purpose of determining a optimal kernel function
6	Test = jackknife(*metaDB*, i);
7	Training = *metaDB*—Test;
8	feaSubset = featureSelection(Training, *fs*);
9	*Training*′ = dimensionalityReduce(Training, feaSubset);
10	*Test*′ = dimensionalityReduce(Test, feaSubset);
11	recModel[*i*] = model(*Training*′, *ml*);
12	recKernel[*i*] = apply recModel[*i*] to *Test*′;
13	recPerformance = evaluate(recKernel, actKernel);
14	**return** recPerformance;

#### Multi-label Evaluation Metrics

For comprehensively evaluating the performance of our kernel recommendation method, three evaluation metrics Hit Rate, *Precision*, and *ARR* are selected.

Let *D* be a multi-label meta-knowledge base consisting of ∣*D*∣ multi-label examples ⟨*x*_*i*_, *Y*_*i*_⟩ (*i* = 1, …, ∣*D*∣), *Y*_*i*_ ⊆ *allKernels* be a kernel identified by Table 3, *R* be our multi-label kernel recommendation method, and $Y_{i}^{\prime }=R(x_{i})$ be the set of labels predicted by *R* for example *x*_*i*_, these three metrics are defined as follows.

- Hit Count (*HC*) and Hit Rate (*HR*)
  Song et al. [62] defined the metrics *HC* and *HR* to evaluate the individual and overall performance of a recommendation model *R* over an example *x*_*i*_ and all examples *D*, respectively.
  $$\begin{array}{c} HC\left(x_{i}\right)=\{\begin{array}{cc} 1 & \text{if}\;Y_{i}^{\prime }\cap Y_{i}\neq \emptyset \\ 0 & \text{otherwise} \end{array} \end{array}$$ (2)
  For an example *x*_*i*_, if the intersection of *Y*_*i*_ and $Y_{i}^{\prime }$ is not empty, then the hit count *HC* is 1, indicating that the recommendation hits the target successfully; otherwise, *HC* is 0, indicating that the recommendation misses.
  $$\begin{array}{c} HR\left(D\right)=\frac{\sum_{i=1}^{N}HC\left(x_{i}\right)}{N}\times 100\% \end{array}$$ (3)
*HR(D)* is the proportion of the examples that are correctly recommended a kernel set among all examples. The greater the value of *HR(D)*, the better the proposed kernel recommendation method.
- Precision
  Precision [81] is used to evaluate the effectiveness of our proposed multi-label kernel recommendation method. This measure calculates the fraction of labels correctly recommended by the multi-label kernel recommendation method, it is defined as follows:
  $$\begin{array}{c} Precision\left(D\right)=\frac{1}{\left|D\right|}\sum_{i=1}^{\left|D\right|}\frac{|Y_{i}\cap Y_{i}^{\prime }|}{|Y_{i}^{\prime }|} \end{array}$$ (4)
- Adjusted ratio of ratios
  ARR defined in Formula Eq 1 is utilized to evaluate the classification performance of SVM with a kernel function recommended by the proposed method.

## Experimental Study

In this section, we experimentally evaluate the proposed multi-label kernel recommendation method over the benchmark data sets.

### Benchmark Data Set

To evaluate our proposed multi-label kernel recommendation method, we collected 132 benchmark data sets from the publicly available repositories UCI, DASL, PROMISE, Agricultural, Agnostic-vs-Prior, and Examples. These data sets cover different fields of life, biology, physical, engineering, and software effect prediction. The brief statistical information of these data sets is given in Table 5.

**Table 5:** Description of the 132 data sets.

ID	Name	Attributes	Instances	Classes	Source
1	anneal	31	898	5	UCI
2	audiology	69	226	24	UCI
3	autos	25	205	6	UCI
4	balance-scale	4	625	3	UCI
5	breast-cancer	9	286	2	UCI
6	breast-w	9	699	2	UCI
7	bridges_version1	11	105	6	UCI
8	bridges_version2	11	105	6	UCI
9	car	6	1728	4	UCI
10	cmc	9	1473	3	UCI
11	colic	22	368	2	UCI
12	credit-a	15	690	2	UCI
13	credit-g	20	1000	2	UCI
14	dermatology	34	366	6	UCI
15	diabetes	8	768	2	UCI
16	ecoli	7	336	8	UCI
17	flags	28	194	8	UCI
18	glass	9	214	6	UCI
19	haberman	3	306	2	UCI
20	hayes-roth_test	3	28	3	UCI
21	hayes-roth_train	4	132	3	UCI
22	heart-c	13	303	2	UCI
23	heart-h	12	294	2	UCI
24	heart-statlog	13	270	2	UCI
25	hepatitis	19	155	2	UCI
26	ionosphere	33	351	2	UCI
27	iris	4	150	3	UCI
28	kdd_synthetic_control	60	600	6	UCI
29	labor	16	57	2	UCI
30	liver-disorders	6	345	2	UCI
31	lung-cancer	56	32	2	UCI
32	lymph	18	148	4	UCI
33	mfeat-fourier	76	2000	10	UCI
34	mfeat-karhunen	64	2000	10	UCI
35	mfeat-morphological	6	2000	10	UCI
36	mfeat-zernike	47	2000	10	UCI
37	molecular-biology_promoters	57	106	4	UCI
38	monks-problems-1_test	6	432	2	UCI
39	monks-problems-1_train	6	124	2	UCI
40	monks-problems-2_test	6	432	2	UCI
41	monks-problems-2_train	6	169	2	UCI
42	monks-problems-3_test	6	432	2	UCI
43	monks-problems-3_train	6	122	2	UCI
44	postoperative-patient-data	8	90	3	UCI
45	primary-tumor	17	339	21	UCI
46	segment	18	2310	7	UCI
47	shuttle-landing-control	6	15	2	UCI
48	solar-flare_1	12	323	2	UCI
49	solar-flare_2	11	1066	3	UCI
50	sonar	60	208	2	UCI
51	soybean	35	683	19	UCI
52	spect_test	22	187	2	UCI
53	spect_train	22	80	2	UCI
54	spectf_test	44	269	2	UCI
55	spectf_train	44	80	2	UCI
56	spectrometer	101	531	48	UCI
57	sponge	44	76	3	UCI
58	tae	5	151	3	UCI
59	tic-tac-toe	9	958	2	UCI
60	vehicle	18	846	4	UCI
61	vote	16	435	2	UCI
62	vowel	13	990	11	UCI
63	wine	13	178	3	UCI
64	zoo	16	101	7	UCI
65	anneal.ORIG	18	898	5	UCI
66	australian	14	690	2	UCI
67	hypothyroid	27	3772	4	UCI
68	kr-vs-kp	36	3196	2	UCI
69	landsat_test	36	2000	6	UCI
70	landsat_train	36	4435	6	UCI
71	mfeat-factors	216	2000	10	UCI
72	mfeat-pixel	240	2000	10	UCI
73	mushroom	21	8124	2	UCI
74	nursery	8	12960	5	UCI
75	optdigits	62	5620	10	UCI
76	page-blocks	10	5473	5	UCI
77	pendigits	16	10992	10	UCI
78	sick	27	3772	2	UCI
79	spambase	57	4601	2	UCI
80	splice	60	3190	3	UCI
81	waveform-5000	40	5000	3	UCI
82	ar3	29	63	2	Promise
83	ar5	29	36	2	Promise
84	usp05-ft	14	72	6	Promise
85	ar1	29	121	2	Promise
86	ar4	29	107	2	Promise
87	ar6	29	101	2	Promise
88	cm1_req	8	89	2	Promise
89	jEdit_4.0_4.2	8	274	2	Promise
90	jEdit_4.2_4.3	8	369	2	Promise
91	jm1	21	10885	2	Promise
92	kc1	21	2109	2	Promise
93	kc1-class-level-binary	86	145	2	Promise
94	kc1-class-level-top5	86	145	2	Promise
95	kc2	21	522	2	Promise
96	kc3	39	458	2	Promise
97	mc1	38	9466	2	Promise
98	mc2	39	161	2	Promise
99	mozilla4	5	15545	2	Promise
100	mw1	37	403	2	Promise
101	pc1	21	1109	2	Promise
102	pc2	36	5589	2	Promise
103	pc3	37	1563	2	Promise
104	pc4	37	1458	2	Promise
105	tae_trainPublic	5	76	3	Examples
106	Balance	3	17	2	DASL
107	Brainsize	6	40	2	DASL
108	Calories	2	40	3	DASL
109	Cars	6	38	6	DASL
110	Eggs	3	48	2	DASL
111	Fiber	4	48	4	DASL
112	FleaBeetles	2	74	3	DASL
113	Fridaythe13th	5	61	12	DASL
114	Hotdogs	2	54	3	DASL
115	LarynxCancer	1	41	2	DASL
116	PopularKids	10	478	2	DASL
117	Pottery	5	26	4	DASL
118	Companies	6	79	9	DASL
119	Michelson	1	100	5	DASL
120	db1-bf	6	63	5	Amirms
121	eucalyptus	19	736	5	Agricultural
122	grub-damage	8	155	4	Agricultural
123	pasture	21	36	3	Agricultural
124	squash-stored	24	52	3	Agricultural
125	squash-unstored	23	52	3	Agricultural
126	white-clover	31	63	4	Agricultural
127	ada_agnostic	47	4562	2	Agnostic-vs-Prior
128	ada_agnostic_train	47	4147	2	Agnostic-vs-Prior
129	ada_agnostic_valid	44	415	2	Agnostic-vs-Prior
130	ada_prior	14	4562	2	Agnostic-vs-Prior
131	ada_prior_train	14	4147	2	Agnostic-vs-Prior
132	ada_prior_valid	14	415	2	Agnostic-vs-Prior

### Experimental Setup

1. Aiming to facilitate the classification of the data sets with Support Vector Machine, we import the LIBSVM tool package [82] into WEKA, in which the *C*-Support Vector Classification is specially designed for dealing with the classification problems. Furthermore, eleven different types of classical kernel functions [30, 83] are chosen as the candidates, including linear, polynomial, radial basis function, sigmoidal function, Laplace, Multiquadric, Rational Quadratic, Spherical, Spline, Wave and Circular.
**Linear kernel function**. If the number of features is large, then it is needless to map the data to a higher dimensional space. That is, using the linear kernel is good enough. The formulation is shown as following:
  $$k(x_{i},x_{j})=x_{i}^{T}\cdot x_{j}.$$
**Polynomial kernel function**. When the number of features is small, one often maps data to higher dimensional spaces. At this time, using nonlinear kernels is a better choice. Polynomial is a kind of nonlinear kernel expressed as following:
  $$k(x_{i},x_{j})={\left(\gamma x_{i}^{T}\cdot x_{j}+coef\right)}^{d},\gamma >0.$$
  Where parameters *γ*, *coef*, *d* need to be initialized, and it is time consuming when the value of degree *d* is large or the training set size is large.
**Radial basis function kernel function (RBF)**. RBF is implemented by using convolutions of the type.
  $$k(x_{i},x_{j})=\exp(-\gamma \|x_{i}-x_{j}{\|}^{2}),\gamma >0.$$
**Sigmoidal kernel function**. The SVM with the Sigmoidal Kernel function is equivalent to the Multi-Layer Perceptron classifier in performance [84].
  $$k(x_{i},x_{j})=\tan(\gamma x_{i}^{T}x_{j}+coef),\gamma >0.$$
**Rational Quadratic Kernel**. The Rational Quadratic Kernel is less computationally intensive than the RBF kernel and can be used as an alternative when using the RBF becomes too expensive.
  $$k(x_{i},x_{j})=1-\frac{\|x_{i}-x_{j}{\|}^{2}}{\|x_{i}-x_{j}{\|}^{2}+c}.$$
**Multiquadric Kernel**. The Multiquadric Kernel is also an example of an non-positive definite kernel and can be used in the same situations as the Rational Quadratic kernel.
  $$k(x_{i},x_{j})=\sqrt{\|x_{i}-x_{j}{\|}^{2}+c^{2}}.$$
**Laplace Kernel**. The Laplace Kernel is less sensitive for changes in the sigma parameter.
  $$k(x_{i},x_{j})=\exp(-\frac{\left\|x_{i}-x_{j}\right\|}{\sigma }).$$
**Circular Kernel**. The Circular Kernel comes from a statistics perspective. It is an example of an isotropic stationary kernel and is positive definite.
  $$k(x_{i},x_{j})=\frac{2}{\pi }\arccos(-\frac{\left\|x_{i}-x_{j}\right\|}{\sigma })-\frac{2}{\pi }\frac{\left\|x_{i}-x_{j}\right\|}{\sigma }\sqrt{1-(\frac{\|x_{i}-x_{j}{\|}^{2}}{\sigma })}\text{,}\;\text{if}\;\|x_{i}-x_{j}\|\;<\;\sigma ,\;\text{zero}\;\text{otherwise}.$$
**Spherical Kernel**. The Spherical Kernel is positive definite.
  $$k(x_{i},x_{j})=1-\frac{3}{2}\frac{\left\|x_{i}-x_{j}\right\|}{\sigma }+\frac{1}{2}{\left(\frac{x_{i}-x_{j}}{\sigma }\right)}^{3},\;\text{if}\;\|x_{i}-x_{j}\|\;<\;\sigma ,\;\text{zero}\;\text{otherwise}.$$
**Wave Kernel**. The Wave Kernel is a symmetric positive semi-definite.
  $$k(x_{i},x_{j})=\frac{\theta }{\left\|x_{i}-x_{j}\right\|}\sin\frac{\left\|x_{i}-x_{j}\right\|}{\theta }.$$
**Spline Kernel**. The Spline Kernel is given as a piece-wise cubic polynomial, as derived in the works [85].
  $$k(x_{i},x_{j})=1+x_{i}\cdot x_{j}+x_{i}\cdot x_{j}\cdot min(x_{i},x_{j})-\frac{x_{i}+x_{j}}{2}\cdot \min{\left(x_{i}\cdot x_{j}\right)}^{2}+\frac{1}{3}\cdot \min{\left(x_{i},x_{j}\right)}^{3}.$$
2. Considering the importance of CPU runtime when evaluating the performance of SVMs with different kernels, we set parameter *β* in Eq 1 to be three values 1%, 10% and 15%, respectively. This allows us to examine the usability of our proposed kernel method under different situations.
3. To produce the meta-knowledge data base, (1) all data characteristics listed in subsection Meta-features are collected as independent variables; (2) the corresponding applicable kernel set is identified for each data set as targets; (3) Friedman test and the post-hoc Holm’s Procedure with the significance *α* = 0.05 are used to guarantee the high confident level.
4. The multi-label kernel recommendation model is built with the help of the java library MULAN, which is specially designed for Multi-Label Learning. Existing multi-label learning methods adopted in our experiments are *BR*, *LP*, *CLR* and *ILC* in the problem transformation level and *ML-KNN* (*K* = 5) in the algorithm adaptation level. Five standard classification algorithms are employed, including IB1, Naive Bayes, J48, Ripper and Random Forest. Although the measures listed in Table 1 are used for characterizing data sets in different perspectives, not all are critical for building multi-label kernel recommendation models. Therefore, we preprocess each data set with feature selection methods *Relief*, *IG* and *CHI*.
5. When evaluating the performance of SVM with different kernels, 10×10-fold cross-validation is applied to guarantee the stability of results and to reduce the effect caused by the order of instances. Meanwhile, the jackknife strategy is employed to recommend kernels for each data set and realize an unbiased estimation for the proposed kernel recommendation method. That is, each data set has an opportunity to be recommended an applicable kernel set and the others are viewed as historical data sets.

### Experimental Results and Analysis

In this section, we compare the performance of our proposed multi-label recommendation method with the single-label recommendation method [62] for kernel selection, the meta-learning based kernel selection method (AliKSM) [30] and the simple multiple kernel learning (MKL) [32] with Polynomial and RBF as the basic kernel function (MKL-Poly) and (MKL-RBF) on the 132 data sets in terms of hit rate (*HR*), *Precision* and *ARR* in Section Recommendation Performance Comparison, respectively. We also analyze the impact of different multi-label classification methods and feature selection methods on our proposed recommendation method in Section Sensitivity Analysis.

#### Recommendation Performance Comparison

The performance of both single-label and multi-label based kernel recommendation models depends on the employed classification algorithms; and many classification algorithms are used for model construction in this paper. In this section, we just present the comparison results of the proposed multi-label kernel recommendation model with the best classification algorithm (Random Forest) and the existing kernel recommendation models in terms of the recommendation hit rate *HR*, *Precision* and the classification performance *ARR*.

Moreover, we also compare the classification performance *ARR* of SVM with the recommended kernel by our proposed multi-label recommendation method to that with (1) the most widely used radial basis function kernel function (RBF) [86], which is the default kernel in LIBSVM [82], and (2) the kernel created by the multiple kernel learning methods *MKL-Poly* and *MKL-RBF*, respectively.

**Fig 2:**
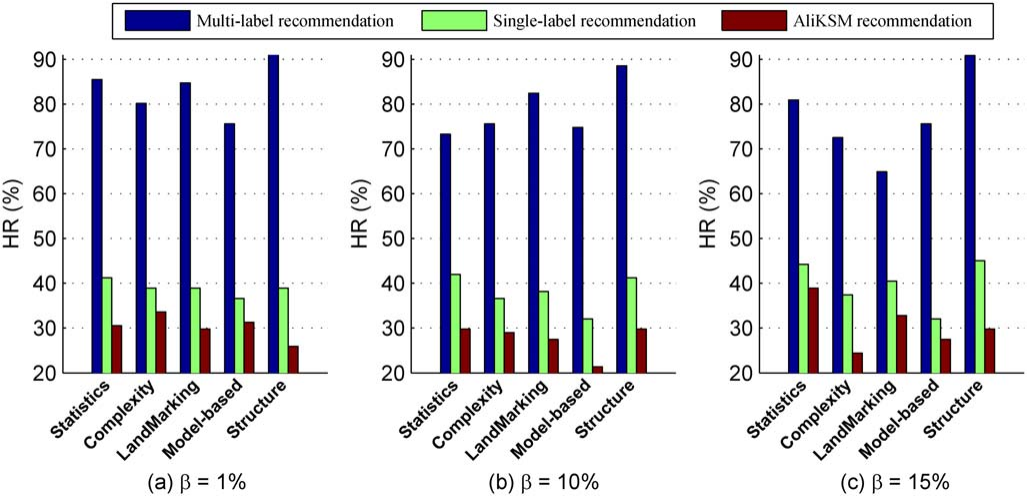
Comparison of different recommendation methods in terms of *HR*.

**Fig 3:**
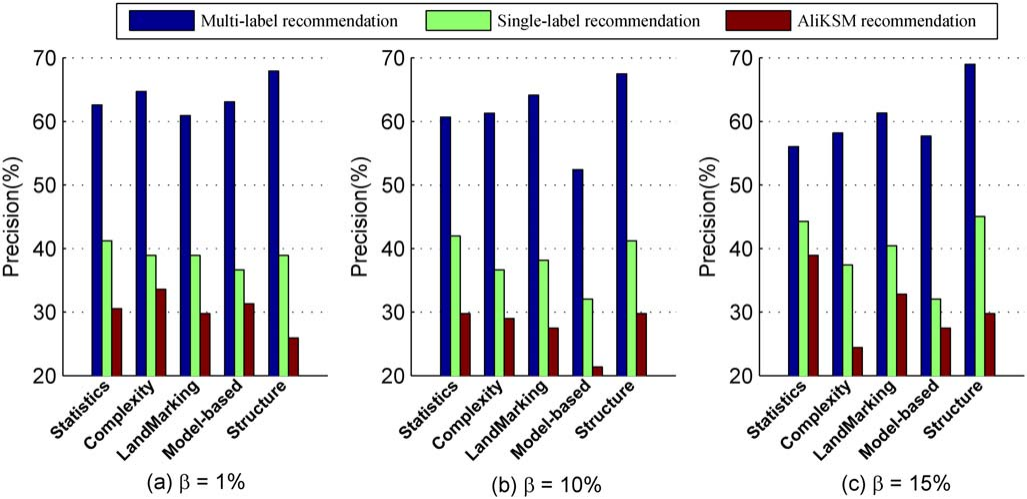
Comparison of different recommendation methods in terms of *Precision*.

**Fig 4:**
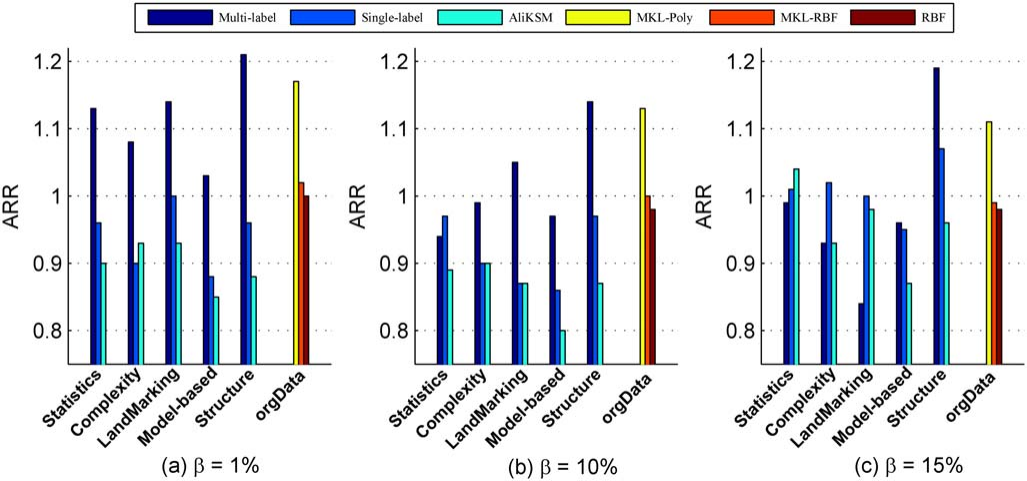
Comparison of different recommendation methods in terms of overall classification performance (*ARR*).

**Fig 5:**
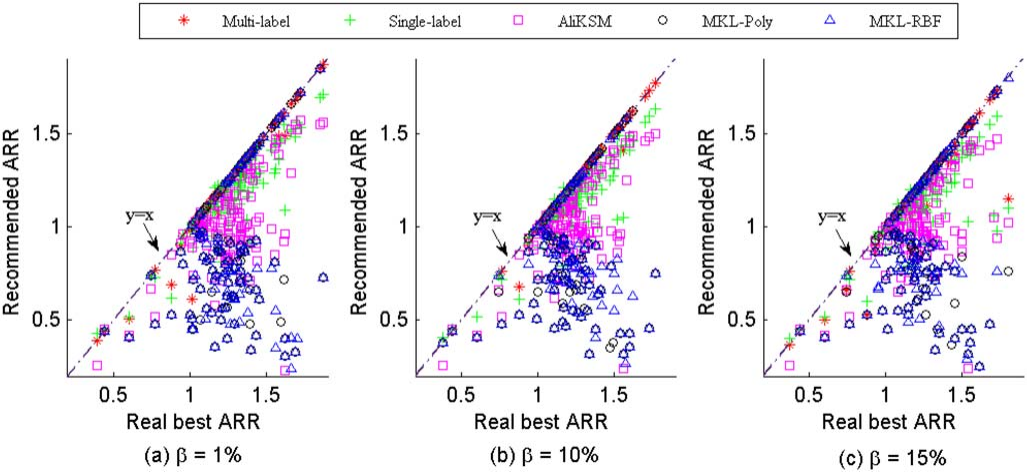
The classification performance (ARR) of SVM with the real best kernel vs. with the recommended kernels.

1. Comparison on hit rate (HR)
Fig 2 shows the recommendation hit rates of the proposed multi-label kernel recommendation method, the single-label recommendation method, the meta-learning based kernel selection method *AliKSM* and the multiple kernel learning methods under *β* = 1%, 10% and 15%, respectively. From this figure we observe that:
  - Whatever the data characteristics and the values of *β* are, the hit rate *HR* of our proposed multi-label kernel recommendation method is significantly greater than those with the other two recommendation methods. Specially, for three values of *β*, the *HR*s of our multi-label recommendations on the structure measures reach up to 91.6%, 88.55% and 90.84%, respectively, which are almost the twice as high as that of other two kernel selection methods. This indicates that our proposed multi-label kernel recommendation method can effectively predict the applicable kernels for the given data sets.
  - The reason why the hit rate *HR* of our proposed multi-label kernel recommendation method is much better than those of the single-label kernel recommendation methods lies that: When constructing the single-label kernel recommendation model with historical data sets, the kernel with the highest classification performance identified by cross validation is selected as the target concept. However, there might exist more than one appropriate kernels for a given data set with no significant differences in the classification performance of SVM. This means single-label kernel recommendation methods miss other applicable kernels, and only if the recommended kernel is the selected one, it hits. This finally results in a lower hit rate.
2. Comparison on precision
Fig 3 shows the recommendation precision of our proposed multi-label kernel recommendation method, the single-label kernel recommendation method and the meta-learning based kernel selection method *AliKSM* with *β* = 1%, 10% and 15%, respectively. From this figure we observe that:
  - The precision of the proposed multi-label kernel recommendation method approaches to 70%, the maximum *Precision*s of both the single-label kernel recommendation method and the automatic kernel selection method *AliKSM* are smaller than the minimum *Precision* of the multi-label kernel recommendation method. It means that our proposed method is more powerful for selecting the applicable kernels for the given data sets.
  - When *β* varies from 1% through 10% to 15%, the *Precision*s of our multi-label kernel recommendation method are all greater than those of the other two recommendation methods by 51.85%, 44.55% and 26.58% at least, respectively. It means whatever the value of *β* is, our proposed method is more effective for kernel selection.
3. Comparison on classification performance (ARR)
Fig 4 shows the ARR of SVM with the kernel functions recommended by different kernel selection methods in terms of *ARR* when *β* = 1%, 10% and 15%, respectively. From this figure we observe that:
  - Whatever the value of *β* is, the classification performance *ARR* of SVM with the kernel recommended by the proposed multi-label recommendation method on the structure measures outperforms those by the other methods.
  - When *β* = 1%, the *ARR*s of SVM with kernel recommended by our proposed method on each kind of meta-features significantly outperform those by other kernel selection methods, except that by the multiple kernel learning method *MKL-Poly*. However, when building the multi-label kernel recommendation model on the structure measures, the classification performance *ARR* of SVM with the kernel recommended by the proposed multi-label recommendation method is still greater than that with the kernel derived from *MKL-Poly* by 3.59%.
  - When *β* = 10%, the *ARR*s of SVM with the kernel recommended by the multi-label recommendation method on most kinds of meta-features are greater than that by the single-label recommendation method by 5.19%–30.46% and that by *AliKSM* by 10.54%–17.42%, respectively. Compared to the multiple kernel learning method *MKL-Poly*, the *ARR* of SVM with the kernel recommended by the multi-label recommendation method on structure measure is improved by 1.05%. Compared to the multiple kernel learning method *MKL-RBF* and the default RBF kernel, the *ARR*s of SVM with the kernel recommended by the multi-label recommendation method are improved by 5.29%, 6.74% on the Landmarking measures and 14.51%, 16.08% on structure measures, respectively.
  - When *β* = 15%, the multi-label kernel recommendation models built on the model-based and structure measures are superior to the single-label recommendation method and the meta-learning based kernel selection method *AliKSM*. The improvements of ARR reach up to 10.43% for the single-label recommendation method and 23.40% for *AliKSM*, respectively. Compared to the multiple kernel learning methods *MKL-Poly*, *MKL-RBF* and the default RBF kernel function, the *ARR* of SVM with the kernel recommended by the multi-label recommendation based on the structure measures is increased by 6.79%, 19.73% and 21.33%, respectively.
  To summarize, with the kernel recommended by our proposed multi-label recommendation method on the structure measures, SVM will obtain the optimal classification performance.
  In Fig 5, a scatter plot is employed to provide an intuitive image on the performance of our proposed kernel recommendation method, the single-label kernel recommendation method, the meta-learning based kernel selection method *AliKSM*, the simple multiple kernel learning methods *MKL-Poly* and *MKL-RBF* for *β* = 1%, 10% and 15%, respectively, where X-axis and Y-axis stand for the classification performance *ARR*s of SVM with the real best kernel and the recommended kernel. The points on the diagonal *y* = *x* mean that the recommendations are optimal. The more the points deviated from the diagonal and the further their distances away from the diagonal, the worse the recommendation performance. From the Fig 5, we observe that:
  - Compared with 59.54%, 57.25% and 54.20% of the recommendations for the single-label recommendation method, 68.70%, 69.47% and 60.31% of the recommendations for *AliKSM*, more than 95% of the recommendations for the multiple learning methods, only 26.72%, 22.90% and 27.48% of the recommendations of our proposed multi-label recommendation method deviated from the diagonal in terms of *ARR* when *β* = 1%, 10% and 15%, respectively. This indicates that the classification performance of SVM with the kernel recommended by the proposed multi-label recommendation method outperforms those with other recommendations and the multi-label recommendation is more likely to recommend the optimal kernels for the given data set.
  - Whatever the value of *β* is, the deviation degree of our proposed method is much smaller than those of the other methods. This means the error of our proposed multi-label kernel recommendation method is much less than those of other methods and the classification performance *ARR*s of SVM with the kernels recommended by our method are most close to the real best ones.
4. Significant test results
  In order to explore whether our multi-label kernel recommendation method is significantly superior to the existing recommendation methods in terms of *HR*, *Precision* and *ARR* when *β* = 1%, 10% and 15%, Wilcoxon signed ranks tests [87, 88] were conducted at the significance level of 0.05 in terms of hit rate *HR*, *Precision* and *ARR*. The alternative hypotheses are that our proposed multi-label kernel recommendation method is better than other methods. Table 6 shows the statistical test results of the proposed multi-label kernel recommendation method vs. the single-label kernel recommendation method, the meta-learning based kernel selection method *AliKSM*, the multiple kernel learning methods *MKL-Poly* and *MKL-RBF* on each kind of meta-features. From Table 6 we observe that:
  - Whatever the value of *β* is, our proposed multi-label kernel recommendation method is significantly superior to the single-label recommendation method and the meta-learning based kernel selection method *AliKSM* on all meta-features in terms of *HR* and *Precision*.
  - When *β* = 1% and 10%, the *ARR* of SVM with the kernel recommended by our multi-label recommendation method obviously outperforms those by the single-label kernel recommendation method and *AliKSM* on most kinds of meta-features. When *β* = 15%, the *ARR*s of SVM with the kernel recommended by the proposed multi-label recommendation method on the model-based and structure measures are significantly greater than those by the single-label recommendation method and *AliKSM*.
  - For each value of *β*, the *ARR* of SVM with the kernel recommended by our multi-label recommendation method on the structure measures significantly outperforms those with the kernel created by the multiple kernel learning methods *MKL-Poly* and *MKL-RBF*.
  In summary, our multi-label kernel recommendation method significantly outperforms the single-label kernel recommendation method and *AliKSM* on most kinds of meta-features in terms of hit rate *HR* and *Precision*. Specially on the structure measures, the classification performance of SVM with the kernel recommended by our multi-label recommendation method is significantly superior to those by all the other methods.

**Table 6:** Statistical test results of performance differences between our method and other methods.

Performance metrics	Data Characteristics	Multi-label vs. Single-label			Multi-label vs AliKSM		
		*β* = 1%	*β* = 10%	*β* = 15%	*β* = 1%	*β* = 10%	*β* = 15%
Hit Rate	Statistics measures	1.16E-13	3.13E-07	9.60E-10	2.37E-19	2.04E-12	4.53E-12
	Complexity measures	1.18E-11	2.36E-10	1.20E-08	3.10E-14	5.08E-14	7.72E-15
	LandMarking measures	2.64E-14	2.70E-13	7.78E-05	2.88E-19	4.56E-19	2.22E-07
	Model-based measures	2.36E-10	4.47E-12	1.80E-12	7.57E-13	5.72E-18	7.72E-15
	Structure measures	4.07E-19	1.16E-15	2.26E-15	4.65E-27	4.52E-22	6.67E-24
Precision	Statistics measures	1.16E-13	3.13E-07	9.60E-10	2.37E-19	2.04E-12	4.53E-12
	Complexity measures	1.18E-11	2.36E-10	1.20E-08	3.10E-14	5.08E-14	7.72E-15
	LandMarking measures	2.64E-14	2.70E-13	7.78E-05	2.88E-19	4.56E-19	2.22E-07
	Model-based measures	2.36E-10	4.47E-12	1.80E-12	7.57E-13	5.72E-18	7.72E-15
	Structure measures	4.07E-19	1.16E-15	2.26E-15	4.65E-27	4.52E-22	6.67E-24
ARR	Statistics measures	2.63E-05	1.22E-01*	5.75E-02*	3.83E-08	1.74E-03	1.05E-02
	Complexity measures	1.46E-03	7.32E-03	6.53E-02*	1.18E-04	2.14E-04	2.79E-03
	LandMarking measures	2.66E-04	5.24E-05	9.18E-01*	1.57E-06	5.77E-07	6.66E-01*
	Model-based measures	5.87E-03	4.02E-03	6.39E-03	5.05E-04	2.95E-05	9.61E-04
	Structure measures	3.41E-08	3.08E-06	1.14E-05	1.48E-12	1.74E-10	5.95E-10
Performance metrics	Data Characteristics	Multi-label vs. MKL-Poly			Multi-label vs MKL-RBF		
		*β* = 1%	*β* = 10%	*β* = 15%	*β* = 1%	*β* = 10%	*β* = 15%
ARR	Statistics measures	5.14E-01*	5.00E-01*	2.38E-01*	1.10E-05	1.65E-02	3.99E-05
	Complexity measures	5.82E-01*	8.61E-01*	9.03E-01*	1.98E-04	2.19E-03	5.43E-03
	LandMarking measures	1.47E-01*	4.03E-01*	7.60E-02*	2.99E-06	5.84E-05	4.13E-01*
	Model-based measures	9.60E-01*	6.55E-01*	9.60E-01*	4.58E-03	1.52E-02	2.90E-03
	Structure measures	2.30E-02	1.88E-02	7.07E-04	4.52E-09	3.78E-09	4.13E-11

* There is no significant difference between the performance of both methods.

In a word, our proposed multi-label kernel recommendation method on the structure measures significantly outperforms other available kernel selection methods. It is capable of recommending the applicable kernels for a new classification problem.

#### Sensitivity Analysis

When building a kernel recommendation model, many multi-label classification methods and feature selection methods can be used. However, different combinations of the multi-label classification methods and feature selection methods will lead to different recommendation models, and further give various recommendations. Therefore, it is necessary to explore which combination is better when constructing the multi-label kernel recommendation model.

**Fig 6:**
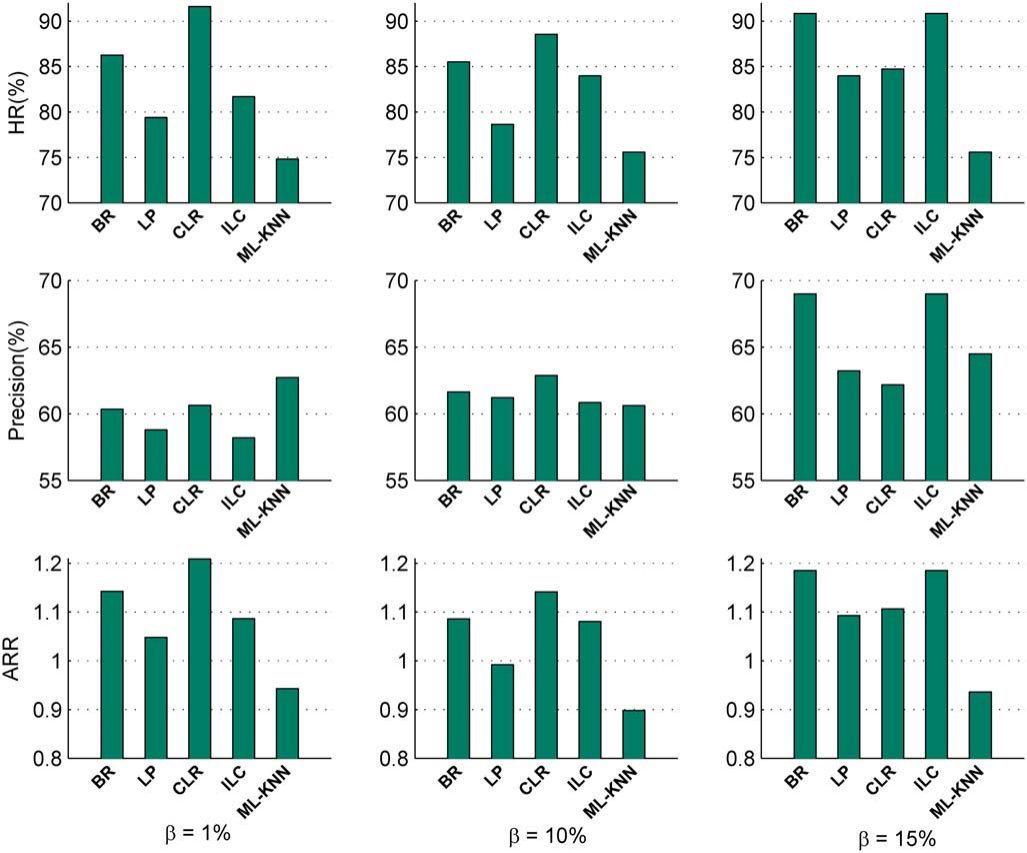
Comparison of different multi-label classification methods in terms of *HR*, *Precision* and *ARR*.

**Fig 7:**
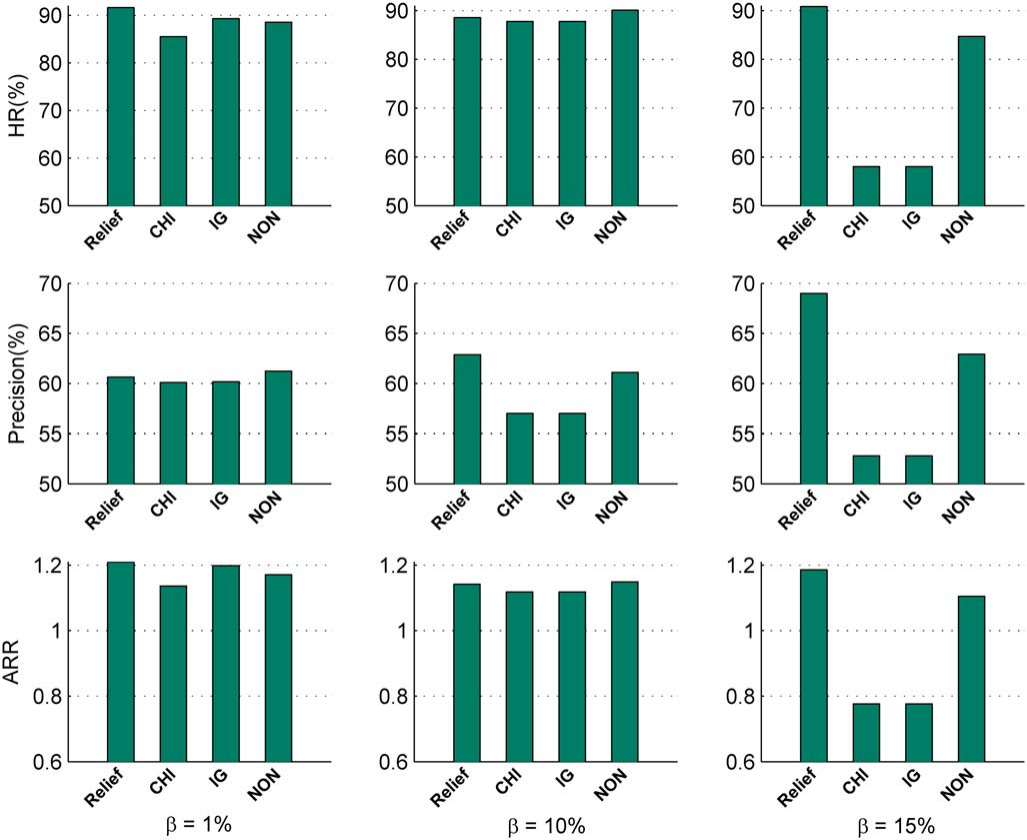
Comparison of different feature selection methods in terms of *HR*, *Precision* and *ARR*.

In this subsection, we analyze the effect of five multi-label classification methods (*BR*, *LP*, *CLR*, *ILC* and *ML-KNN* (*k* = 5)) and three representative feature selection methods (*Relief*, *CHI* and *IG*) on the recommendations for the 132 data sets in terms of the average *HR*, *Precision* and *ARR* for three different *β* values. Figs 6 and 7 illustrates the sensitivity analysis results of multi-label classification methods and feature selection methods, respectively.

1. Effect of multi-label classification methods
  From Fig 6, we observe that:
  - For all the three *β* values, the average recommendation hit rate *HR*, *Precision* and the classification performance *ARR* vary with different multi-label classification methods. It indicates that different multi-label classification methods do affect the performance of the multi-label kernel recommendation method.
  - When *β* = 1% and 10%, the multi-label recommendation methods with *CLR* obtain the best performance. When *β* = 15%, the multi-label recommendation methods with *BR* and *ILC* equally achieve the optimal *HR*, *Precision* and *ARR*.
2. Effect of feature selection methods
  From Fig 7, we can observe that:
  - For each of the three *β* values, the performance of the multi-label kernel recommendation method vary with different feature selection methods. It means that different feature selection methods indeed affect the performance of the multi-label kernel recommendation method.
  - When *β* = 1%, employing *Relief* and *IG* for feature selection, the recommendations with the multi-label recommendation method outperforms those with *CHI* and without feature selection (*NON*). When *β* = 10%, there is no significant difference between the multi-label methods with feature selection and without feature selection (*NON*) in terms of *HR* and *ARR*. It is worth noting that the recommendation precision with *Relief* is greater than those with *NON*, *CHI* and *IG* by 2.92%, 10.33% and 10.33%, respectively. When *β* = 15%, the performance of the multi-label kernel recommendation method with *Relief* outperforms those with *NON*, *CHI* and *IG* by 7.21%, 56.58% and 56.58% in terms of *HR*, 9.67%, 30.67% and 30.67% in terms of *Precision*, and 7.27%, 52.62%, 52.62% in terms of *ARR*, respectively.
  Overall, we learn about that the combination of the multi-label classification method CLR and the feature selection method *Relief* is benefit for optimizing the multi-label kernel recommendation method.

## Conclusion

Aiming to identify the applicable kernels for SVM for a new classification problem, in this paper, we have presented a multi-label learning based kernel recommendation method.

In our method, all available data characteristics are first extracted from each data set as the meta-features and the really applicable kernels are identified via cross-validation in terms of the relative performance metric integrating the classification success ratio with the CPU time. Then, the relationship is built on the meta-knowledge data base consisted of the meta-features and the multi-label target with the multi-label classification method. After that, the applicable kernels are recommended for a new classification problem according to its data characteristics.

For the purpose of confirming the effectiveness of our proposed recommendation method, we conducted the experiments on 132 public data sets and compared the proposed multi-label kernel recommendation method with other kernel selection methods in terms of hit rate and precision on all types of data characteristics. Moreover, we also evaluated the classification performance of SVM with the kernel functions recommended by different selection methods and that with the most widely used RBF kernel function. The experimental results demonstrate that our proposed multi-label kernel recommendation method outperforms the other kernel selection methods, and it can comprehensively and precisely identify the applicable kernels for a new classification problem. We also carried out the sensitivity analysis for the multi-label kernel recommendation method so as to observe under which situation the proposed method performs better, in which we respectively analyzed the effect from different multi-label classification methods and feature selection methods on our recommendation results. After that, we draw the conclusions that (1) the multi-label classification method *CLR* is a better choice for constructing the multi-label kernel recommendation method when *β* = 1% and 10%, while *BR* and *ILC* is better when *β* = 15%, and (2) the feature selection method *Relief* is more effective for improving the performance of our kernel recommendation method.

For the further work, we plan to study the parameter optimization or the combination of multiple applicable kernels for SVM based on multi-label learning.

## References

[1] Boser BE, Guyon IM, Vapnik VN. A training algorithm for optimal margin classifiers In: Proceedings of the fifth annual workshop on Computational learning theory. ACM; 1992 p. 144–152.

[2] Cortes C, Vapnik V. Support-vector networks. Machine learning. 1995;20(3):273–297. 10.1023/A:1022627411411

[3] Hsu CW, Lin CJ. A comparison of methods for multiclass support vector machines. IEEE Transactions on Neural Networks. 2002;13(2):415–425. 10.1109/72.991427 18244442

[4] Nguyen MN, Rajapakse JC. Multi-class support vector machines for protein secondary structure prediction. Genome Informatics. 2003;14:218–227. 15706536

[5] Vapnik VN, Chervonenkis AY. On the uniform convergence of relative frequencies of events to their probabilities. Theory of Probability & Its Applications. 1971;16(2):264–280. 10.1137/1116025

[6] Vapnik V, Kotz S. Estimation of dependences based on empirical data. Springer; 2006.

[7] Tong S, Koller D. Support vector machine active learning with applications to text classification. The Journal of Machine Learning Research. 2002;2:45–66.

[8] Leopold E, Kindermann J. Text categorization with support vector machines. How to represent texts in input space? Machine Learning. 2002;46(1–3):423–444.

[9] Zhang W, Yoshida T, Tang X. Text classification based on multi-word with support vector machine. Knowledge-Based Systems. 2008;21(8):879–886. 10.1016/j.knosys.2008.03.044

[10] Byun H, Lee SW. Applications of support vector machines for pattern recognition: A survey In: Pattern recognition with support vector machines. Springer; 2002 p. 213–236.

[11] Ayat NE, Cheriet M, Suen CY. Automatic model selection for the optimization of SVM kernels. Pattern Recognition. 2005;38(10):1733–1745. 10.1016/j.patcog.2005.03.011

[12] Abe S. Support vector machines for pattern classification. vol. 53 Springer; 2005.

[13] Shi Z, Han M. Support vector echo-state machine for chaotic time-series prediction. IEEE Transactions on Neural Networks. 2007;18(2):359–372. 10.1109/TNN.2006.885113 17385625

[14] Sapankevych NI, Sankar R. Time series prediction using support vector machines: a survey. IEEE Computational Intelligence Magazine. 2009;4(2):24–38. 10.1109/MCI.2009.932254

[15] Brown MP, Grundy WN, Lin D, Cristianini N, Sugnet CW, Furey TS, et al Knowledge-based analysis of microarray gene expression data by using support vector machines. Proceedings of the National Academy of Sciences. 2000;97(1):262–267. 10.1073/pnas.97.1.262 PMC26651 10618406

[16] Furey TS, Cristianini N, Duffy N, Bednarski DW, Schummer M, Haussler D. Support vector machine classification and validation of cancer tissue samples using microarray expression data. Bioinformatics. 2000;16(10):906–914. 10.1093/bioinformatics/16.10.906 11120680

[17] Akay MF. Support vector machines combined with feature selection for breast cancer diagnosis. Expert systems with applications. 2009;36(2):3240–3247. 10.1016/j.eswa.2008.01.009

[18] Bradford JR, Westhead DR. Improved prediction of protein-protein binding sites using a support vector machines approach. Bioinformatics. 2005;21(8):1487–1494. 10.1093/bioinformatics/bti242 15613384

[19] Aronszajn N. Theory of reproducing kernels. Transactions of the American mathematical society. 1950;p. 337–404.

[20] Heckman N, et al The theory and application of penalized methods or Reproducing Kernel Hilbert Spaces made easy. Statistics Surveys. 2012;6:113–141. 10.1214/12-SS101

[21] Smola AJ, Schölkopf B. On a kernel-based method for pattern recognition, regression, approximation, and operator inversion. Algorithmica. 1998;22(1–2):211–231. 10.1007/PL00013831

[22] Brereton RG, Lloyd GR. Support vector machines for classification and regression. Analyst. 2010;135(2):230–267. 10.1039/B918972F 20098757

[23] Vapnik V, Chapelle O. Bounds on error expectation for support vector machines. Neural computation. 2000;12(9):2013–2036. 10.1162/089976600300015042 10976137

[24] Muller KR, Mika S, Ratsch G, Tsuda K, Scholkopf B. An introduction to kernel-based learning algorithms. IEEE Transactions on Neural Networks. 2001;12(2):181–201. 10.1109/72.914517 18244377

[25] Cawley GC. Leave-one-out cross-validation based model selection criteria for weighted LS-SVMs In: International Joint Conference on Neural Networks. IEEE; 2006 p. 1661–1668.

[26] Bach FR, Lanckriet GR, Jordan MI. Multiple kernel learning, conic duality, and the SMO algorithm In: Proceedings of the twenty-first international conference on Machine learning. ACM; 2004 p. 6–13.

[27] Zien A, Ong CS. Multiclass multiple kernel learning In: Proceedings of the 24th international conference on Machine learning. ACM; 2007 p. 1191–1198.

[28] Thadani K, Jayaraman V, Sundararajan V. Evolutionary selection of kernels in support vector machines In: International Conference on Advanced Computing and Communications. IEEE; 2006 p. 19–24.

[29] Sullivan KM, Luke S. Evolving kernels for support vector machine classification In: Proceedings of the 9th annual conference on Genetic and evolutionary computation. ACM; 2007 p. 1702–1707.

[30] Ali S, Smith-Miles KA. A meta-learning approach to automatic kernel selection for support vector machines. Neurocomputing. 2006;70(1):173–186. 10.1016/j.neucom.2006.03.004

[31] Wang W, Guo J, Men C. An approach for kernel selection based on data distribution In: Rough Sets and Knowledge Technology. Springer; 2008 p. 596–603.

[32] Rakotomamonjy A, Bach F, Canu S, Grandvalet Y. SimpleMKL. Journal of Machine Learning Research. 2008;9:2491–2521.

[33] Gönen M, Alpaydin E. Multiple kernel learning algorithms. The Journal of Machine Learning Research. 2011;12:2211–2268.

[34] Zhang ML, Zhou ZH. A Review On Multi-Label Learning Algorithms. IEEE Transactions on Knowledge and Data Engineering. 2013;26(8):1819–1837. 10.1109/TKDE.2013.39

[35] Tsoumakas G, Katakis I. Multi-label classification: An overview. International Journal of Data Warehousing and Mining. 2007;3(3):1–13. 10.4018/jdwm.2007070101

[36] Tsoumakas G, Katakis I, Vlahavas I. Mining multi-label data In: Data mining and knowledge discovery handbook. Springer; 2010 p. 667–685.

[37] Yang S, Kim SK, Ro YM. Semantic home photo categorization. IEEE Transactions on Circuits and Systems for Video Technology. 2007;17(3):324–335. 10.1109/TCSVT.2007.890829

[38] Qi GJ, Hua XS, Rui Y, Tang J, Mei T, Zhang HJ. Correlative multi-label video annotation In: Proceedings of the 15th international conference on Multimedia. ACM; 2007 p. 17–26.

[39] Katakis I, Tsoumakas G, Vlahavas I. Multilabel text classification for automated tag suggestion. In: ECML PKDD Discovery Challenge. vol. 18; 2008 p. 75.

[40] Thabtah FA, Cowling P, Peng Y. MMAC: A new multi-class, multi-label associative classification approach In: Fourth IEEE International Conference on Data Mining. IEEE; 2004 p. 217–224.

[41] Zhu S, Ji X, Xu W, Gong Y. Multi-labelled classification using maximum entropy method In: Proceedings of the 28th annual international ACM SIGIR conference on Research and development in information retrieval. ACM; 2005 p. 274–281.

[42] Gopal S, Yang Y. Multilabel classification with meta-level features In: Proceedings of the 33rd international ACM SIGIR conference on Research and development in information retrieval. ACM; 2010 p. 315–322.

[43] Wang G, Song Q, Xueying Z. A Generic Multi-label Learning Based Classification Algorithm Recommendation Method. ACM Transactions on Knowledge Discovery from Data. 2014;9(1):1–31. 10.1145/2629474

[44] Brazdil PB, Soares C, Da Costa JP. Ranking learning algorithms: Using IBL and meta-learning on accuracy and time results. Machine Learning. 2003;50(3):251–277. 10.1023/A:1021713901879

[45] Staelin C. Parameter selection for support vector machines. Hewlett-Packard Company, Tech. Rep. HPL-2002-354R1; 2003.

[46] Zhang D, Chen S, Zhou ZH. Learning the kernel parameters in kernel minimum distance classifier. Pattern Recognition. 2006;39(1):133–135. 10.1016/j.patcog.2005.08.001

[47] Zhang D, hua Zhou Z, Chen S. Adaptive Kernel Principal Component Analysis with Unsupervised Learning of Kernels. In: IEEE International Conference on Data Mining; 2006. p. 1178–1182.

[48] Lin CJ, Hsu CW, Chang CC. A practical guide to support vector classification; 2010. Available: http://www.csie.ntu.edu.tw/cjlin/. Accessed 2010 April 15.

[49] Sonnenburg S, Rätsch G, Schäfer C, Schölkopf B. Large scale multiple kernel learning. The Journal of Machine Learning Research. 2006;7:1531–1565.

[50] Howley T, Madden MG. The genetic kernel support vector machine: Description and evaluation. Artificial Intelligence Review. 2005;24(3–4):379–395. 10.1007/s10462-005-9009-3

[51] Partridge M, Jabri M. Robust principal component analysis In: Proceedings of the 2000 IEEE Signal Processing Society Workshop. vol. 1 IEEE; 2000 p. 289–298.

[52] Kalousis A, Gama J, Hilario M. On data and algorithms: Understanding inductive performance. Machine Learning. 2004;54(3):275–312. 10.1023/B:MACH.0000015882.38031.85

[53] Ali S, Smith KA. On learning algorithm selection for classification. Applied Soft Computing. 2006;6(2):119–138. 10.1016/j.asoc.2004.12.002

[54] Brazdil P, Gama J, Henery B. Characterizing the applicability of classification algorithms using meta-level learning In: Machine Learning. Springer; 1994 p. 83–102.

[55] Lindner G, Studer R. AST: Support for algorithm selection with a CBR approach In: Principles of Data Mining and Knowledge Discovery. Springer; 1999 p. 418–423.

[56] Castiello C, Castellano G, Fanelli AM. Meta-data: Characterization of input features for meta-learning In: Modeling Decisions for Artificial Intelligence. Springer; 2005 p. 457–468.

[57] Ho TK, Basu M. Complexity measures of supervised classification problems. IEEE Transactions on Pattern Analysis and Machine Intelligence. 2002;24(3):289–300. 10.1109/34.990132

[58] Bensusan H, Giraud-Carrier C. Discovering task neighbourhoods through landmark learning performances In: Principles of Data Mining and Knowledge Discovery. Springer; 2000 p. 325–330.

[59] Pfahringer B, Bensusan H, Giraud-Carrier C. Tell me who can learn you and i can tell you who you are: Landmarking various learning algorithms. In: Proceedings of the 17th International Conference on Machine Learning; 2000. p. 743–750.

[60] Duin RP, Pekalska E, Tax DM. The characterization of classification problems by classifier disagreements In: Proceedings of the 17th International Conference on Pattern Recognition. vol. 1 IEEE; 2004 p. 141–143.

[61] Pavel YPPAF, Soares BC. Decision Tree-Based Data Characterization for Meta-Learning. IDDM. 2002;p. 111.

[62] Song Q, Wang G, Wang C. Automatic recommendation of classification algorithms based on data set characteristics. Pattern recognition. 2012;45(7):2672–2689. 10.1016/j.patcog.2011.12.025

[63] Huang J, Ling CX. Using AUC and accuracy in evaluating learning algorithms. IEEE Transactions on Knowledge and Data Engineering. 2005;17(3):299–310. 10.1109/TKDE.2005.50

[64] Nakhaeizadeh G, Schnabl A. Development of Multi-Criteria Metrics for Evaluation of Data Mining Algorithms. In: Proceedings of the Third International Conference on Knowledge Discovery and Data Mining; 1997. p. 37–42.

[65] Jensen DD, Cohen PR. Multiple comparisons in induction algorithms. Machine Learning. 2000;38(3):309–338. 10.1023/A:1007631014630

[66] Friedman M. The use of ranks to avoid the assumption of normality implicit in the analysis of variance. Journal of the American Statistical Association. 1937;32(200):675–701. 10.1080/01621459.1937.10503522

[67] Friedman M. A comparison of alternative tests of significance for the problem of m rankings. The Annals of Mathematical Statistics. 1940;11(1):86–92. 10.1214/aoms/1177731944

[68] Holm S. A simple sequentially rejective multiple test procedure. Scandinavian journal of statistics. 1979;6(2):65–70.

[69] Fürnkranz J, Hüllermeier E, Mencía EL, Brinker K. Multilabel classification via calibrated label ranking. Machine Learning. 2008;73(2):133–153. 10.1007/s10994-008-5064-8

[70] Clare A, King RD. Knowledge discovery in multi-label phenotype data In: Principles of data mining and knowledge discovery. Springer; 2001 p. 42–53.

[71] Schapire RE, Singer Y. BoosTexter: A boosting-based system for text categorization. Machine learning. 2000;39(2–3):135–168. 10.1023/A:1007649029923

[72] Zhang M, Zhou Z. ML-KNN: A lazy learning approach to multi-label learning. Pattern recognition. 2007;40(7):2038–2048. 10.1016/j.patcog.2006.12.019

[73] Elisseeff A, Weston J. A kernel method for multi-labelled classification. In: NIPS. vol. 14; 2001 p. 681–687.

[74] Zhang M, Zhou Z. Multilabel neural networks with applications to functional genomics and text categorization. IEEE Transactions on Knowledge and Data Engineering. 2006;18(10):1338–1351. 10.1109/TKDE.2006.162

[75] Crammer K, Singer Y. A family of additive online algorithms for category ranking. The Journal of Machine Learning Research. 2003;3:1025–1058.

[76] Freund Y, Schapire RE. A decision-theoretic generalization of on-line learning and an application to boosting. Journal of computer and system sciences. 1997;55(1):119–139. 10.1006/jcss.1997.1504

[77] Yu K, Yu S, Tresp V. Multi-label informed latent semantic indexing In: Proceedings of the 28th annual international ACM SIGIR conference on Research and development in information retrieval. ACM; 2005 p. 258–265.

[78] Boutell MR, Luo J, Shen X, Brown CM. Learning multi-label scene classification. Pattern recognition. 2004;37(9):1757–1771. 10.1016/j.patcog.2004.03.009

[79] Yang Y, Pedersen JO. A comparative study on feature selection in text categorization. In: International Conference on Machine Learning. vol. 97; 1997 p. 412–420.

[80] Kira K, Rendell LA. A practical approach to feature selection In: Proceedings of the ninth international workshop on Machine learning. Morgan Kaufmann Publishers; 1992 p. 249–256.

[81] Godbole S, Sarawagi S. Discriminative methods for multi-labeled classification In: Advances in Knowledge Discovery and Data Mining. Springer; 2004 p. 22–30.

[82] Chang CC, Lin CJ. LIBSVM: a library for support vector machines. ACM Transactions on Intelligent Systems and Technology. 2011;2(3):27 10.1145/1961189.1961199

[83] Genton MG. Classes of Kernels for Machine Learning: A Statistics Perspective. Journal of Machine Learning Research. 2001;2:299–312.

[84] Cybenko G. Approximation by superpositions of a sigmoidal function. Mathematics of control, signals and systems. 1989;2(4):303–314. 10.1007/BF02551274

[85] Wolpert DH, Macready WG. Support Vector Machines for Classification and Regression. UK: University of Southampton, School of Electronics and Computer Science, ISIS technical report; 1998.

[86] Vapnik VN, Vapnik V. Statistical learning theory. vol. 1 Wiley New York; 1998.

[87] Wilcoxon F. Individual comparisons by ranking methods. Biometrics. 1945;1(6):80–83. 10.2307/3001968

[88] Demšar J. Statistical comparisons of classifiers over multiple data sets. The Journal of Machine Learning Research. 2006;7:1–30.

